# Leveraging FinTech for positive ESG outcomes through regional innovation: insights from a knowledge capital perspective

**DOI:** 10.3389/fpubh.2025.1641241

**Published:** 2025-09-01

**Authors:** Xinrui Sun, Guo Wu

**Affiliations:** ^1^Faculty of Engineering Sciences, University College London, London, United Kingdom; ^2^Shengxiang Business School, Sanda University, Shanghai, China

**Keywords:** financial technology, regional ESG, sustainable development, regional innovation, urbanization, income disparity, knowledge capital

## Abstract

Financial technology (FinTech) is an increasingly important driver of sustainable development, with a crucial role in influencing environmental, social, and governance (ESG) outcomes that underpin public health and well-being. In this study, we theoretically and empirically examine the impact of FinTech on regional ESG performance. Our empirical analysis is based on a panel dataset covering 31 Chinese provinces from 2011 to 2023. We provide evidence that FinTech enhances regional ESG performance, primarily by promoting regional innovation. Drawing on a tentative knowledge capital model, we show that the innovation-enhancing effect is achieved by improving innovation efficiency and reducing innovation costs related to sustainable practices. Furthermore, the positive impact of FinTech on regional ESG performance is more pronounced in regions with lower income disparity and higher urbanization. These findings highlight the need for differentiated FinTech policies, tailored to local socio-economic and environmental conditions, to effectively support ESG goals, foster regional sustainable development, and ultimately contribute to improved public health and well-being.

## 1 Introduction

As global economic development enters a new phase, sustainable development has become a focus of attention for policymakers and academics in various countries (1). Financial technology (FinTech) as an innovative force, it can integrate digital technology with financial services. It also offers novel pathways to support global transition toward sustainability (2). Many governments are starting to focus on environmental, social, and governance (ESG) performance at both corporate and regional levels and they regard ESG performance as a key metric for sustainable development (3). It could be beneficial for regions that effectively use FinTech to enhance their ESG profiles and also handle environmental challenges in the rapidly evolving digital financial landscape (4). However, the underlying dynamics that FinTech development affects how well regions perform on ESG measures are still underexplored. Within the context of China, regions can be very different in their economic growth and how much they use new technology (5). As regions gradually integrate FinTech solutions, it is crucial to have a clear understanding of how these initiatives translate into tangible ESG improvements and foster sustainable regional development. To bridge this research gap, we utilize provincial-level evidence from China to examine the impact of FinTech on regional ESG performance.

The understanding of how FinTech drives regional ESG performance can be vital for various reasons. First, as economies strive for comprehensive sustainability, regions must reconcile economic development objectives with enhanced environmental and social outcomes. FinTech’s potential in enhancing financial efficiency and inclusion (6) as well as promoting green finance initiatives (7) may offer actionable insights for policymakers tasked with this complex mandate, particular for China and other nations committed to ambitious national sustainable development goals (8). Second, China has many regions with different economic setups and varying levels of readiness for new technology. This could potentially make it even more urgent to find effective ways to ensure that technological innovation also helps improve ESG performance (9). While current research on FinTech is extensive, deficiencies still exist at the regional ESG level. Many studies focus on the impact of FinTech on corporate ESG performance (10, 11), or explore its socio-environmental performance at the national level (4). However, quantitative research on how FinTech influences regional-level ESG performance through the channel of regional innovation, and the theoretical foundation of such mechanism remain insufficient. On the other hand, although the effect of FinTech in mitigating information friction has received attention (12), its further implications on the heterogeneous effects on ESG under different regional conditions has not been fully explored.

In this study, we specifically examine the impact of FinTech development on regional ESG performance across Chinese provinces and the underlying dynamics that drive this impact. We utilize the panel data from 31 Chinese provinces during 2011 and 2023. We employ a two-way fixed effects model and conduct a series of robustness checks to validate our hypotheses. In order to elucidate how FinTech can improve regional innovation and ultimately improve regional ESG outcomes, we further construct a tentative theoretical model from a knowledge capital perspective. We theoretically show that that enhancing regional innovation efficiency and reducing regional innovation costs would eventually lead to the enhancement of regional ESG performance. We also investigate the heterogeneous impact of FinTech in regions with different urban–rural income gaps and urbanization levels. Our study contributes to the existing literature on three grounds. First, we supplement the existing literature that primarily focuses on the corporate level by providing regional-level evidence regarding the impact of FinTech on ESG performance. Second, we conduct theoretical and empirical analyses on how regional innovation acts as a channel. Third, we elucidate the heterogeneous role of key regional socio-economic factors, including the urban–rural income gap and urbanization level.

The rest of this article is structured as follows. Section 2 reviews the literature on different aspects of FinTech, ESG performance, and regional innovation. Section 3 elaborates on the theoretical analysis, the proposed mechanisms, and the research hypotheses. Section 4 details the econometric model specification, variable definitions and measurement methods, and data sources. Section 5 discusses the empirical results, including baseline regressions, a series of robustness checks, channel tests, and heterogeneity analysis. Section 6 concludes the research and proposes corresponding policy implications.

## 2 Literature review

The existing literature has indicated the positive promotional effect of financial technology (FinTech) on corporate environmental, social, and governance (ESG) performance from various perspectives. Du et al. (10) propose that FinTech development can significantly enhance corporate ESG performance and validate this hypothesis by using firm-level data from China, and the authors also identify the channel of alleviating financing constraints. Naysary and Shrestha (13) employ a wavelet-DCC GARCH method and identify a significant positive relationship between FinTech and ESG markets, with the correlation being time-varying and exhibiting mean-reverting characteristics. Wang and Esperança (14) conduct a survey study and show that digital transformation positively affects ESG performance through the mediating variable of market performance, with digital innovation culture playing a positive moderating role in the paths from digital adoption to corporate competitiveness and from digital adoption to digital management. From an external attention perspective, Li et al. (15) find that external attention moderates the relationship between FinTech and green innovation, with this mechanism being more pronounced in state-owned enterprises (SOEs) or firms with excellent ESG performance. Gao et al. (11) employ the entropy method to construct a FinTech development index and reveal that FinTech improves corporate ESG performance via promoting green innovation and alleviating financial misallocation. Ding et al. (16) identify that FinTech can promote corporate ESG practices by reducing the cost of equity and increasing equity and short-term loan issuance. As a fundamental technology driving FinTech development, artificial intelligence (AI) has been shown to significantly improve corporate ESG performance by Huang et al. (17), who use the China’s introduction of ‘*National New Generation Artificial Intelligence Innovation Development Pilot Zones*’ policy as a quasi-natural experiment. The authors show that AI pilot policies significantly improve corporate ESG performance through green technology innovation and research investment levels. Agboare et al. (18) utilize a panel vector autoregression (PVAR) method to analyze 122 Chinese A-share financial institutions and discover that technology-driven financial disruption significantly boosts ESG performance. Liu et al. (19) further find that FinTech can break down information barriers, optimize investment structures, and stimulate green innovation. Interestingly, the authors also note that executives with political backgrounds are more effective in leveraging FinTech development to improve ESG practices.

On the other hand, macro-level studies also reveal the importance of regional characteristics and international differences with respect to the impact of FinTech on ESG. Chueca Vergara and Ferruz Agudo (20) broadly review the nexus between FinTech and sustainability and argue that FinTech can make overall financial operations more sustainable by promoting green finance. Mertzanis (4) posits that FinTech has an impact on socio-environmental performance, while Trotta et al. (21) further explore the nexus between FinTech and ESG through a bibliometric lens, emphasizing FinTech’s significant role in ESG disclosure, corporate governance, and sustainability. Tran and Le (22) provide a country case study and conclude that enterprises located in cities with highly developed FinTech could exhibit better ESG performance. Recent studies further support the existence of regional heterogeneity and show that the positive effects of digital finance on ESG are stronger in areas with larger economies and well-developed digital infrastructure (23). The impact on innovation follows a clear geographical pattern, being most effective in China’s eastern and central regions (24, 25). Furthermore, Khan et al. (26) argue that blockchain technology and green innovation technology can positively affect ESG sustainability performance, with global financial integration likely playing an important role. The authors posit that this effect is significant in both developed and emerging economies. In addition, innovation is likely to be an important channel through which FinTech boosts regional capabilities. This is suggested by Chin et al. (27), and the authors show that innovation could act as a crucial mediator in driving outcomes like green finance. Besides, interestingly, from a firm life-cycle perspective, Hu et al. (28) find that the impact of FinTech on ESG performance is most significant during the decline stage, followed by maturity and growth stages, with green finance playing the strongest moderating role during the growth stage.

It can be seen that the existing literature studies consistently suggest that FinTech positively impacts corporate ESG through mechanisms like eased financing, better information disclosure, green innovation promotion, and optimized investments. We also know from the literature that the effect of FinTech on ESG can be moderated by firm, industry, and life-cycle factors. However, empirical validation of FinTech’s effect on regional ESG performance still remains scarce. Meanwhile, the significant role of regional innovation in the FinTech-ESG relationship has been overlooked. In addition, examinations of regional heterogeneity frequently default to broad geographical categorizations, overlooking important socio-economic moderators like economic development, income levels, industrial structure, and urbanization. To address these deficiencies, we employ a provincial panel data model to empirically investigate FinTech’s effect on regional ESG performance in China. Our findings aim to inform tailored FinTech policies aligned with regional characteristics.

## 3 Hypothesis development

We first explore the direct impact of FinTech on regional ESG performance. According to the study by (29), FinTech can improve financial service delivery, enhance resource allocation efficiency, and optimize risk management. From an innovation theory viewpoint, Almaqtari et al., (30) argue that emerging technologies like big data, blockchain, and artificial intelligence can promote sustainable economic and social development. Implied by transaction cost economics (31), FinTech could contribute through an information friction and transaction cost reduction effect. FinTech utilizes digital payments, online credit, and smart investment platforms to significantly lower information asymmetry and transaction costs in the market. Quintiliani (32) discusses that, by mitigating these frictions, FinTech makes funding more accessible for micro, small, and medium-sized enterprises and green projects, thereby alleviating financing constraints and potentially enhancing ESG performance. Based on this, we propose our first hypothesis:

*H1: FinTech can significantly enhance regional ESG performance.*

Second, FinTech can play a crucial role in promoting technological innovation and enhancing R&D efficiency (33). At the technological innovation level, FinTech encourages enterprises and wider regional systems to increase investment in green technology R&D, optimize risk allocation, and boost overall innovation output. This role is strongly implied by endogenous growth theory (34, 35), suggesting that technological progress and knowledge accumulation are key internal drivers of sustained economic development. By acting as a catalyst to improve innovation process efficiency, FinTech likely facilitates more impactful ESG-related advancements and fosters the endogenous growth of regional sustainable capabilities. Meanwhile, the sustainable development theory advocates for meeting present needs without compromising the welfare of future generations (36). Under this framework, FinTech channels more social capital toward energy conservation, emission reduction, pollution control, and ecological restoration projects through innovative instruments like green bonds and green funds. It enhances financial inclusion for low-income and remote populations through digital inclusive finance, promoting social equity. This leads to our second hypothesis:

*H2: There is an innovation-enhancing channel through which FinTech improves regional ESG performance.*

Furthermore, leveraging big data analytics and intelligent risk control, FinTech may optimize the management of environmental and social risks to achieve coordinated economic, social, and environmental development. Building on this, the regional economic growth theory explores how factors such as capital, technology, resources, and policies operate across different regions and their impact on economic disparities (37). With the rise of FinTech, this theory has expanded to incorporate a digital finance perspective (38). FinTech enhances the efficiency of capital and information flows both within and between regions, promoting industrial digital transformation and the formation of green industrial clusters. Simultaneously, technology spillovers and agglomeration economies strengthen regional competitiveness. However, differences in economic development levels, resident income, industrial depth, and urbanization rates across regions lead to significant spatial heterogeneity in FinTech’s promotion effect on regional ESG performance and green growth (39). Accordingly, we propose the following hypothesis:

*H3: There are heterogeneous impacts in areas with differences in the disposable income gap between urban and rural residents and the level of urbanization.*

## 4 Model and data

### 4.1 Model specification

We employ a two-way fixed effects (TWFE) model to identify the impact of FinTech development on regional ESG performance, as shown in Equation 1. The TWFE model controls both province fixed effects and time fixed effects. On one hand, we posit that each China’s province possesses unique characteristics in culture, history, and economic structure, which may remain constant across different time points and are unobservable. By eliminating such individual heterogeneity, we ensure an accurate estimation of the true impact of FinTech on ESG performance. On the other hand, time fixed effects capture time trends and common time-related shocks affecting the dependent variable.


(1) $$\mathrm{ES}G_{\mathrm{it}}=\beta_{0}+\beta_{1}\text{FinTec}h_{\mathrm{it}}+\beta_{2}\text{Control}s_{\mathrm{it}}+\mu_{i}+\lambda_{t}+\varepsilon_{\mathrm{it}}$$

The term ESG*_it_* denotes the level of regional ESG performance, FinTech*_it_* denotes the level of FinTech development, Controls*_it_* is a set of control variables, the term *μ_i_* represents province fixed effects, *λ_t_* represents time fixed effects, and the term *ε_it_* is the error term.

### 4.2 Variables

#### 4.2.1 Dependent variable

**Figure 1:**
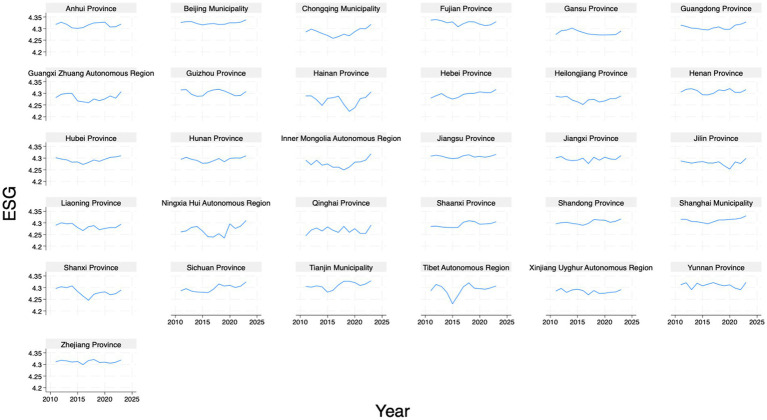
ESG performance across provinces.

Following the existing studies (11, 40, 41), we adopt the ‘*Huazheng ESG Score*’ to reflect the level of regional ESG performance. The variable *ESG* is primarily calculated based on data publicly disclosed by listed companies, as well as other sources such as financial reports, corporate social responsibility (CSR) reports, sustainability reports, announcements from regulatory authorities, and media reports. According to publicly available information, the Huazheng ESG evaluation system adopts a three-tier indicator structure. Details can be found at the official website.^1^ The regional ESG performance is obtained by weighting the ESG levels of listed companies within the province according to their enterprise size. Figure 1 shows the ESG performance across the sample.

#### 4.2.2 Independent variable

**Figure 2:**
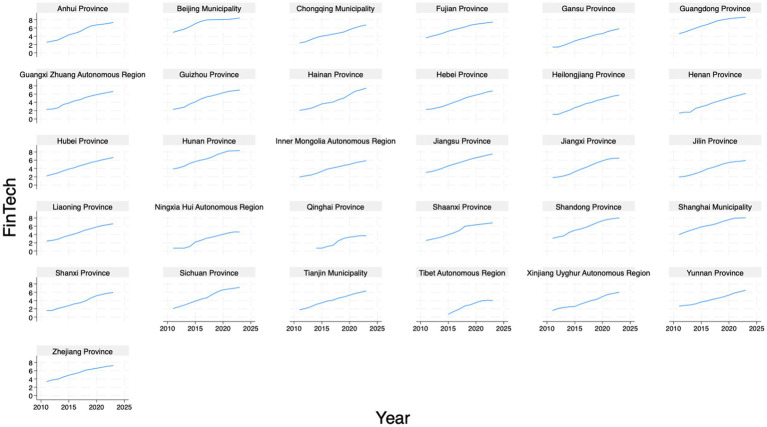
FinTech levels across provinces.

We use the logarithmic value of the number of registered FinTech companies plus one, to measure the degree of FinTech development. The data are obtained from the China’s ‘*Tianyancha*’ enterprise information query platform. Specifically, we search for keywords (in Chinese) including ‘*Financial Technology*’, ‘*Artificial Intelligence*’, ‘*Cloud Computing*’, ‘*Big Data*’, ‘*Blockchain*’, and ‘*Internet of Things*’, to identify relevant companies in normal operation, then match and count them by province. We leverage the count of technology-intensive firms at the regional level to capture the supply-side capacity underpinning FinTech development. This approach directly quantifies the concentration of specialized entities, including startups and innovators whose core focus is FinTech-enabling technologies like AI, Big Data, and Cloud Computing. The FinTech-enabling technologies form the foundational ecosystem essential for China’s unique FinTech dynamism. We posit that the number of registered FinTech companies in a province can well reflect the activity level and market size of the regional FinTech industry. A higher number usually indicates a stronger FinTech market, attracting more enterprises and talent, thereby driving the region’s FinTech development. Figure 2 provides the FinTech levels across provinces. It can be observed that FinTech has shown a remarkable development trend in all provinces in recent years.

#### 4.2.3 Control variables

According to the existing studies (16, 19, 26, 28), we include the following control variables. First, we include gross domestic product per capita (variable name: *GDP*) to reflect the degree of per capita regional economic development. Economically developed regions generally have stronger fiscal and technological capacities, allowing for more investments in environmental protection, social welfare, and improved governance structures. Second, we use population density (variable name: *Population*). Population density reflects regional resource demand and social structure. Densely populated areas may face greater pressure in social welfare and environmental governance, leading to more proactive ESG efforts by governments and enterprises. It also affects the efficient allocation of resources and overall consumption levels. Specifically, we calculate population density by dividing the year-end permanent resident population (in 10,000 s) by land area (in 10,000 square kilometers). Third, we think that fiscal budget expenditures (variable name: *Fiscal*) are likely linked to regional ESG performance, as government support often determines investment capacity in green technology, social programs, and governance initiatives. We adopt the ratio of general fiscal budget expenditures to regional GDP to measure the level of fiscal support. Fourth, the upgrading of industrial structure (variable name: *IndStr*, as represented by the ratio of tertiary to secondary industry output), may directly affect regional environmental and social performance. A higher share of the tertiary industry (typically associated with low-resource and low-emission services) may enhance social welfare and environmental quality, whereas heavy reliance on the secondary industry, especially heavy industries, may increase environmental governance burdens. Fifth, we also include social consumption (variable name: *Consumption*) to account for its potential impact on regional ESG performance. Higher consumption levels might imply stronger environmental awareness and social responsibility, as well as stronger market demand, prompting more proactive ESG measures from enterprises and governments. We use the ratio of total retail sales of consumer goods to regional GDP to measure the level of social consumption. Overall, by controlling for these variables, we aim to eliminate potential confounding factors and more accurately identify the impact of FinTech development on regional ESG performance.

### 4.3 Data and descriptive statistics

The final sample contains 31 Chinese provinces from the year 2011 to 2023. All variables are drawn from various authoritative sources, including the iFinD database for corporate-level information, the National Bureau of Statistics for national indicators, provincial statistical yearbooks for local data, and the China Statistical Yearbook. Together, these sources provide a comprehensive, reliable foundation for our further analyses. Table 1 presents the descriptive statistics. It can be seen that, over 403 province-year observations, the regional ESG score has a mean of 4.2946 with a standard deviation of 0.0201, indicating that provincial ESG performance is generally high and narrowly dispersed. FinTech development averages 4.6520 (SD = 1.8507), reflecting cross-province differences in FinTech industry activity. Per capita GDP, used to proxy economic development, has a mean value of 10.8915 (SD = 0.4715), suggesting moderate variation in wealth levels. Population density averages 5.3442 (SD = 1.4470) and highlights observable heterogeneity in demography. Fiscal support has a mean of 0.2441 (SD = 0.1307), and this reflects some differences in government capacity. The industrial structure variable has a value of 0.8353 on average (SD = 0.2371), while social consumption averages 0.3280 (SD = 0.0481) and the result suggests relatively consistent consumer spending across provinces.

**Table 1:** Descriptive statistics.

Variable	Observations	Mean	SD	Min	Max
ESG	403	4.2946	0.0201	4.2227	4.3380
FinTech	396	4.6520	1.8507	0.6931	8.6294
GDP	403	10.8915	0.4715	9.6819	12.2075
Population	403	5.3442	1.4470	1.2565	8.2821
Fiscal	403	0.2441	0.1307	0.0999	0.8560
IndStr	403	0.8353	0.2371	0.4233	1.9006
Consumption	403	0.3280	0.0481	0.1652	0.4763

SD, standard deviation; Min, minimum; Max, maximum; All statistics above are based on the logarithmic transformation of the original direct measures.

Table 2 reports the correlation coefficients among *FinTech*, *ESG*, and the set of control variables. Overall, *FinTech* is positively correlated with ESG performance (0.3893), suggesting that the development of financial technology may contribute to improved regional ESG outcomes. *FinTech* also shows strong positive correlations with economic development (*GDP*, 0.7746) and industrial upgrading (*IndStr*, 0.4655), indicating that provinces with higher levels of economic development and more advanced industrial structures tend to have greater FinTech capabilities. *GDP* is positively correlated with population density (*Population*, 0.4982), while fiscal support (*Fiscal*) is significantly negatively correlated with *Population* (−0.8010). Additionally, *FinTech* is negatively correlated with *Fiscal* (−0.4145), implying that provinces with stronger fiscal support may lag in FinTech advancement. Overall, the positive correlation between *FinTech* and *ESG*, along with the close relationships between economic development, industrial structure, fiscal support, and ESG outcomes, provides initial insights into the interplay among these variables. Table 2 serves as a basis for understanding the relationships among them.

**Table 2:** Correlation matrix.

Variable	ESG	FinTech	GDP	Population	Fiscal	IndStr	Consumption
ESG	1.0000						
FinTech	0.3893	1.0000					
GDP	0.4167	0.7746	1.0000				
Population	0.4729	0.4399	0.4982	1.0000			
Fiscal	−0.3711	−0.4145	−0.3986	−0.8010	1.0000		
IndStr	0.1103	0.4655	0.4589	0.1971	0.1272	1.0000	
Consumption	0.1289	0.0283	−0.0330	0.2742	−0.1500	0.0305	1.0000

All variables are continuous and winsorized at 1% and 99% percentiles; Pearson correlation coefficients are reported.

Table 3 reports the variance inflation factor (VIF) and 1/VIF for each explanatory variable. VIF is used to assess the presence of multicollinearity among variables. Generally, a higher VIF indicates a stronger linear relationship between one variable and the others, which may signal potential multicollinearity issues. We observe that all explanatory variables have VIF values below the commonly accepted threshold of 10, and this suggests the absence of severe multicollinearity. The mean VIF value is 2.7896, indicating that multicollinearity is not a serious concern in this study. It can be seen that the VIF test results suggest that the specified econometric model is unlikely to be significantly affected by multicollinearity.

**Table 3:** Variance inflation factor (VIF) and 1/VIF of variables.

Variable	VIF	1/VIF
Fiscal	3.9186	0.2552
Population	3.8215	0.2617
FinTech	2.9983	0.3335
GDP	2.8691	0.3485
IndStr	1.9801	0.5050
Consumption	1.1497	0.8698
Mean VIF	2.7896	

## 5 Empirical results

### 5.1 Baseline regression

Table 4 presents the coefficient estimates of *FinTech*. Referring to Column (4), it is evident that the development of FinTech enhances overall performance in environmental, social, and governance dimensions. This finding supports Hypothesis 1. From a resource-based view (RBV) perspective, FinTech improves financial accessibility and enables firms to allocate more resources toward ESG initiatives (42). Meanwhile, enhanced digital financing reduces transaction costs (43), likely facilitating investments in green technologies and socially responsible projects.

**Table 4:** Baseline regression results.

Variables	Dependent variable: ESG			
	(1)	(2)	(3)	(4)
FinTech	0.0042^***^	0.0016^**^	0.0025	0.0052^**^
	(0.0005)	(0.0008)	(0.0023)	(0.0025)
GDP		0.0095^***^	−0.0013	−0.0045
		(0.0031)	(0.0107)	(0.0153)
Population		0.0059^***^	−0.0083	0.0143
		(0.0012)	(0.0256)	(0.0232)
Fiscal		0.0134	−0.1060^**^	−0.0443
		(0.0145)	(0.0417)	(0.0420)
IndStr		−0.0143^***^	−0.0276^**^	−0.0109
		(0.0050)	(0.0133)	(0.0139)
Consumption		0.0117	−0.0175	0.0070
		(0.0189)	(0.0213)	(0.0235)
Constant	4.2753^***^	4.1564^***^	4.3967^***^	4.2599^***^
	(0.0025)	(0.0318)	(0.1627)	(0.2123)
Province FE	No	No	Yes	Yes
Year FE	No	No	No	Yes
*N*	396	396	396	396
Adjusted *R*^2^	0.149	0.290	0.607	0.692

We report standard errors in parentheses (*p < 0.1, **p < 0.05, ***p < 0.01); Province fixed effects and year fixed effects are controlled; Control variables are GDP, Population, Fiscal, IndStr, and Consumption. In economics literature, this is a normal practice. *,**, and *** represent significance levels.

Specifically, in column (1), the coefficient of *FinTech* is 0.0042 (*p* < 0.01), suggesting a significant positive effect of FinTech on ESG. However, since no control variables or fixed effects are included, this result cannot serve as solid evidence. As shown in column (2), after incorporating more control variables, the coefficient of FinTech remains significant (*p* < 0.05), though fixed effects are still not accounted for. Column (3) reports the result controlling only for province fixed effects, where the coefficient becomes insignificant. Column (4) provides the estimation that includes both control variables and province and year fixed effects. The coefficient of *FinTech* is 0.0052 and remains significant at the 5% level, indicating that FinTech exerts a significantly positive impact on regional ESG performance after accounting for various fixed effects.

### 5.2 Robustness checks

#### 5.2.1 Variable substitution method

First, we substitute the FinTech variable to re-estimate the impact of FinTech development on regional ESG performance. Drawing on the research of Zhang et al. (44) and Jagtiani and Lemieux (52), we adopt a word frequency analysis method to measure the level of FinTech development in a firm. Specifically, we use key search terms including ‘*Big Data*’, ‘*Cloud Computing*’, ‘*Artificial Intelligence*’, ‘*Blockchain*’, ‘*Biometrics*’, ‘*Online Payment*’, ‘*Mobile Payment*’, ‘*Third-party Payment*’, ‘*Peer-to-peer Lending*’, ‘*Online Loans*’, ‘*Online Banking*’, and ‘*Open Banking*’. We adopt the Baidu Indices of such terms and further utilize the entropy method to assign weights to each index, finally generating the provincial-level FinTech data, as an alternative measure *FinTech_a*. As shown in column (1) of Table 5, the impact of FinTech development on regional ESG performance remains significantly positive.

**Table 5:** Robustness check (variable substitution).

Variables	Dependent variable: ESG		
	(1)	(2)	(3)
FinTech_a	0.0145^*^		
	(0.0085)		
FinTech		0.0052^*^	0.0016^**^
		(0.0026)	(0.0008)
GDP	−0.0097	−0.0045	0.0095^***^
	(0.0163)	(0.0148)	(0.0031)
Population	−0.0008	0.0143	0.0059^***^
	(0.0254)	(0.0139)	(0.0012)
Fiscal	−0.0409	−0.0443	0.0134
	(0.0432)	(0.0273)	(0.0144)
IndStr	−0.0052	−0.0109	−0.0143^***^
	(0.0143)	(0.0108)	(0.0050)
Consumption	0.0188	0.0070	0.0117
	(0.0250)	(0.0196)	(0.0187)
Constant	4.3046^***^	4.2704^***^	4.1564^***^
	(0.2280)	(0.1432)	(0.0315)
Province Fixed Effects	Yes	Yes	Yes
Time Fixed Effects	Yes	Yes	Yes
*N*	372	396	396
Adjusted *R*^2^	0.684	–	–

We report standard errors in parentheses (*p < 0.1, **p < 0.05, ***p < 0.01); Province fixed effects and year fixed effects are controlled; The column (2) provides the estimation results using Driscoll-Kraay standard errors (45), which account for autocorrelation and heteroscedasticity; The column (3) provides the estimation results using maximum likelihood estimation (MLE), and the log likelihood value is 1057.9181.

#### 5.2.2 Changes in estimation method

Driscoll-Kraay (DK) standard errors are specifically designed to handle heteroscedasticity, serial correlation, and cross-sectional correlation in panel data (45). In practical panel data analysis, these issues often co-occur, and traditional standard error estimation methods may fail to adequately address them, leading to inaccurate results. Column (2) of Table 5 reports the estimation results using DK standard errors, showing that the positive effect of FinTech on regional ESG standards remains significant. Besides, in comparison to OLS estimation, maximum likelihood estimation (MLE) is based on maximizing the likelihood function. MLE can be used to estimate parameters under various error term distributions, such as normal, *t*-distribution, or non-normal distributions, making it more flexible in dealing with heteroscedasticity, serial correlation, or non-normal error terms. As shown in column (3) of Table 5, the positive impact of FinTech on ESG remains robust under the MLE estimation method.

#### 5.2.3 Endogeneity treatments

To mitigate potential endogeneity concerns, we employ a dynamic panel data model using the generalized method of moments (GMM) estimation method (46, 47). The dynamic panel model essentially uses a set of lagged variables as instruments for estimation. Specifically, we adopt the system GMM approach to examine the relationship between FinTech and regional ESG performance. As shown in Table 6, Column (1) reports the results of the one-step System GMM, while Column (2) presents the two-step System GMM results. Both estimations control for province and year fixed effects. In both cases, the coefficient of FinTech is positive and statistically significant at the 5% level, indicating that the development of FinTech contributes to the improvement of regional ESG performance. The term L. ESG is the one-period lagged variable of ESG. The system GMM is a widely used estimation technique for dynamic panel data models, especially suitable for datasets with lagged dependencies and endogeneity issues (47). It is interesting to note that the lagged variable L. ESG also has a positive coefficient, suggesting that past regional ESG performance has a significant influence on the current level. Additionally, both the AR (2) test and the Hansen test are not statistically significant, indicating no second-order autocorrelation and no problem with the validity of instrumental variables, thereby confirming the reliability of the model estimates. Overall, we provide empirical evidence for the role of FinTech in promoting regional ESG performance.

**Table 6:** System GMM estimation results.

Variables	Dependent variable: ESG	
	(1) One-step system GMM	(2) Two-step system GMM
L. ESG	0.6745^***^	0.6733^***^
	(0.0752)	(0.0851)
FinTech	0.0016^**^	0.0016^*^
	(0.0007)	(0.0009)
GDP	0.0046	0.0044
	(0.0033)	(0.0039)
Population	0.0016	0.0018
	(0.0010)	(0.0012)
Fiscal	0.0113	0.0133
	(0.0140)	(0.0153)
IndStr	−0.0070	−0.0074
	(0.0057)	(0.0065)
Consumption	−0.0099	−0.0128
	(0.0141)	(0.0148)
Constant	1.3382^***^	1.3457^***^
	(0.2929)	(0.3374)
Province Fixed Effects	Yes	Yes
Time Fixed Effects	Yes	Yes
*N*	367	367
AR(2) *p*-value	0.663	0.688
Hansen test *p*-value	0.900	0.900

We report standard errors in parentheses (*p < 0.1, **p < 0.05, ***p < 0.01); L. ESG represents the first-order lag of the ESG variable.

### 5.3 Further discussion

#### 5.3.1 The innovation-enhancing effect

As proposed in section 3, FinTech may not only directly enhance regional ESG performance but also exert an indirect influence by stimulating regional innovation—an effect we term the ‘*innovation-enhancing effect*’. To elucidate this mechanism more clearly at the regional level, we develop a tentative theoretical model from a knowledge capital perspective. We aim to reveal how regional FinTech development fosters the accumulation of ESG-related innovation capabilities, which in turn improves overall regional ESG performance. The theoretical underpinnings of this model draw from endogenous growth theory and transaction cost economics. First, we consider that the level of regional FinTech development (*F_t_*) at time *t* shall incorporate a range of technological innovations that have demonstrated significant economic value (29). As shown in Equation 2, we propose that a region maximizes its long-term ESG welfare *W* by choosing an optimal level of investment in ESG-related innovation *I_t_*. The regional objective function is specified as:


(2) $$W=\int_{0}^{\infty }e^{-\mathrm{\rho t}}[\mathrm{ESG}(K_{t})-C(I_{t},F_{t})]\mathrm{dt}$$

where *ρ* is the social discount rate. The regional ESG performance ESG(*K_t_*) is dependent on the stock of regional ESG innovation capacity *K_t_*. The cost of ESG innovation investment C(*I_t_*, *F_t_*) is dependent on the regional FinTech level *F_t_*. The dynamics of regional ESG innovation capacity (*K_t_*) are governed by:


(3) $${\dot{K}}_{t}=I_{t}^{\alpha }F_{t}^{\beta }-{\mathrm{\delta K}}_{t}$$

The tern ${\dot{K}}_{t}$ represents the accumulation of knowledge capital, which stands for innovation capacity. The parameter *α* (0 < *α* < 1) is the elasticity of innovation output with respect to investment. The crucial parameter *β* (*β* > 0) captures FinTech’s direct efficiency-enhancing effect on regional innovation production. A higher *F_t_* makes regional innovation investment more productive. As suggested by the endogenous growth theory (34, 35), technological advancements can shift the innovation production function, enabling more efficient knowledge creation. Existing literature also provides evidence that FinTech can promote green innovation, a key component of ESG capability (11, 15, 19). The parameter *δ* represents the depreciation rate of the knowledge. The cost of regional ESG innovation investment C(*I_t_*, *F_t_*) is specified as:


(4) $$C(I_{t},F_{t})=\frac{1}{2}\phi I_{t}^{2}{F_{t}}^{-\gamma }$$

where *ϕ* is a cost parameter. The term ${F_{t}}^{-\gamma }$ (with γ > 0) signifies FinTech’s cost-reducing effect on regional innovation investment. According to transaction cost economics (31), FinTech can lower the overall social costs of these investments by reducing information asymmetry, search costs, and the financing costs of ESG projects. Finally, regional ESG performance, ESG(*K_t_*), as can be seen in Equation 5, is a function of the accumulated knowledge:


(5) $$\mathrm{ESG}(K_{t})=AK_{t}^{\theta }$$

where the term *A* is a productivity parameter, and *0 < θ < 1* is the elasticity of ESG performance with respect to knowledge. Subsequently, employing the Hamiltonian method, the model yields an optimal path for ESG-related innovation investment *I_t_∗*. In the steady state, where *K_t_* = 0 and the shadow price of *K_t_* is stable, the regional ESG innovation capacity (*K^∗^*) and overall ESG performance (*ESG*^∗^) are determined. As shown in Equation 6, the steady-state *K^∗^* is then simplified as:


(6) $$K^{*}(F)=M(A,\alpha ,\theta ,\phi ,\rho ,\delta )\times F^{\frac{\alpha \gamma +2\beta }{2-\alpha \theta }}$$

Where the term M(A,α,θ,ϕ,ρ,δ) is related to multiple parameters, and the steady-state regional ESG performance can then be expressed as *ESG^∗^(F) = A*(*K^∗^*(*F*))*^θ^*. The elasticity of steady-state regional ESG performance with respect to the FinTech development level (*F*) is:


(7) $$\eta_{\mathrm{ESG},F}=\frac{\partial \ln\mathrm{ES}G^{*}}{\partial \ln F}=\frac{\theta (\alpha \gamma +2\beta )}{2-\alpha \theta }$$

**Figure 3:**
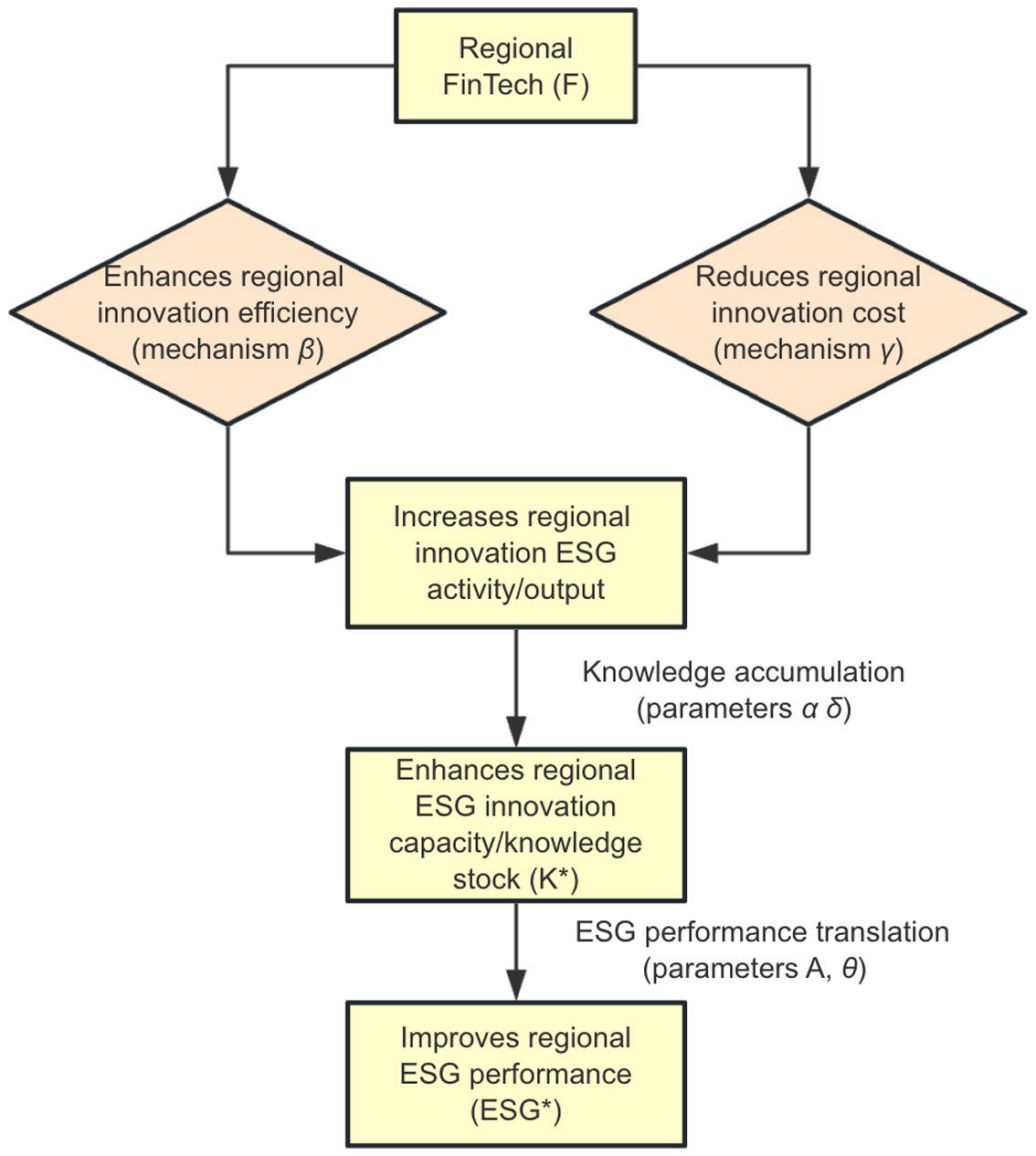
Mechanisms of the innovation-enhancing effect.

The Equation 7 shows that the total elasticity of regional ESG performance with respect to FinTech (as represented by the term *η_ESG, F_*) is positive. This positive sign clearly indicates that an increase in the regional FinTech development level (*F*) indeed leads to an improvement in overall regional ESG performance (ESG^*^). This overall positive impact is realized through two underlying mechanisms embedded in the model. First is the regional innovation efficiency channel. In our theoretical model, this is primarily manifested through the positive effect of parameter *β*. According to Equation 3, FinTech can directly enhance the productive efficiency of regional investments in ESG-related innovation, as shown in the model’s equation for the accumulation of regional ESG innovation capacity. This implies that for any given level of regional ESG investment, a higher level of FinTech development can promote a greater effective output of ESG-related new knowledge and capabilities. The accelerated generation speed and improved quality of this overall regional innovation capacity *K^∗^*, translate directly into better ESG outcomes. The logic of this channel aligns with the perspectives of the endogenous growth theory, which posits that advanced technologies, acting as catalysts for economic growth, drive sustainable development by fostering more effective knowledge accumulation. Numerous findings in the literature regarding FinTech’s promotion of green innovation also corroborate the contribution of this efficiency-enhancing effect to ESG goals. As revealed by the research of Gao et al. (11) and Liu et al. (19), FinTech can enhance ESG performance by stimulating and promoting green innovation, while the study by Huang et al. (17) also indicates that technologies like artificial intelligence can improve ESG by advancing green technology innovation. These findings confirm the positive role of FinTech in enhancing regional innovation efficiency. Second, there is a regional innovation cost reduction mechanism. The specification of parameter *γ* in the model reflects FinTech’s positive impact in this aspect. The development of FinTech contributes to systematically reducing the effective costs of undertaking ESG-related innovation and investment within a region, as shown in Equation 4. FinTech can lessen the overall socioeconomic burden of promoting regional ESG development by optimizing resource allocation, providing more convenient financing channels, or reducing information acquisition costs (48). A reduction in costs can incentivize the region to undertake a higher level of optimal ESG investment (*I_t_^∗^*), thereby promoting the formation of a larger stock of regional ESG innovation capacity (*K^∗^*) and consequently improving final ESG performance. This mechanism is consistent with the core ideas of transaction cost economics, which suggests that technological progress can enhance the feasibility and attractiveness of economic activities by reducing market frictions, search costs, and financing barriers. Existing research also indicates that the development of regional FinTech can indeed support corporate and regional ESG practices by, for example, alleviating financing constraints. The study by Du et al. (10) points out that FinTech can alleviate financing constraints, which aligns with the mechanism in this model where FinTech lowers innovation costs. Concurrently, Chueca Vergara and Ferruz Agudo (20) also emphasize FinTech’s role in promoting green finance, which can be understood as lowering the financing threshold and costs for green innovation projects, thereby driving regional sustainable development. The theoretical model shows how FinTech influences regional ESG performance via the innovation-enhancing effect, as shown by the flowchart in Figure 3. This figure clearly demonstrates how FinTech impacts regional ESG innovation activities and capacity accumulation through two sub-mechanisms: the innovation efficiency enhancement (*β* mechanism) and the innovation cost reduction (γ mechanism), ultimately transmitting to regional ESG performance.

#### 5.3.2 Heterogeneity analysis

The preceding analysis explores the overall impact of FinTech on regional ESG performance. This section delves deeper into how FinTech functions under different environmental conditions—specifically, how it drives ESG performance in regions with low-income disparity and low urbanization levels. With respect to the urban–rural disposable income gap, as shown in Table 7, FinTech has a significantly positive effect on ESG in the low-income-gap group, with a coefficient of 0.0097, significant at the 1% level. However, in the high-income-gap group, the coefficient of FinTech is 0.0047 and not statistically significant. These results suggest that FinTech has a more pronounced effect on ESG performance in regions with smaller urban–rural income disparities. One possible explanation is that smaller income gaps imply a more equitable distribution of financial resources, allowing residents and businesses in rural areas to better access and benefit from FinTech. As a result, the positive effect of FinTech on ESG is more evident in these areas. Moreover, from the perspective of innovation diffusion theory, regions with smaller income gaps often enjoy better information dissemination efficiency. Consequently, rural populations and enterprises are more likely to access and adopt FinTech innovations, which in turn promotes overall regional innovation and enhances ESG performance. Further, as shown in columns (3) and (4), the coefficients of FinTech on ESG performance are 0.0102 and 0.0132, respectively. The current finding supplements the viewpoint that FinTech may be leveraged to enhance urban development (49) and support environmental sustainability (50). The positive role of urbanization in fostering sustainable development is also highlighted by Zheng et al. (51), who find that the urbanization rate is a significant positive predictor of regional sustainable and renewable energy development. On one hand, highly urbanized areas tend to have more developed financial service infrastructure. As a supplement to traditional financial systems, FinTech can more efficiently enhance ESG-related corporate practices in these regions. Enterprises in highly urbanized areas also tend to have greater acceptance and capability in applying FinTech, making its impact on ESG performance more substantial. On the other hand, the development and application of FinTech are highly dependent on robust digital infrastructure, such as high-speed internet and mobile payment networks. However, low-urbanization areas often lag in building such infrastructure, which may hinder the widespread adoption and effectiveness of FinTech.

**Table 7:** Results of heterogeneity analysis.

Variables	Dependent variable: ESG			
	Income cap between urban and rural residents		Urbanization level	
	(1) Low	(2) High	(3) Low	(4) High
FinTech	0.0097^***^	0.0047	0.0102^**^	0.0132^***^
	(0.0036)	(0.0043)	(0.0045)	(0.0035)
GDP	−0.0031	0.0129	−0.0041	0.0344
	(0.0234)	(0.0236)	(0.0252)	(0.0258)
Population	0.0154	0.0451	0.0361	0.0429
	(0.0268)	(0.0521)	(0.0467)	(0.0345)
Fiscal	−0.2055^***^	0.0065	−0.0009	−0.1542^**^
	(0.0590)	(0.0668)	(0.0773)	(0.0611)
IndStr	0.0069	−0.0244	−0.0295	0.0225
	(0.0173)	(0.0286)	(0.0280)	(0.0204)
Consumption	−0.0344	0.0425	0.0193	−0.0161
	(0.0337)	(0.0372)	(0.0380)	(0.0353)
_cons	4.2365^***^	3.9168^***^	4.1367^***^	3.6036^***^
	(0.2934)	(0.3832)	(0.3869)	(0.3345)
Province Fixed Effects	Yes	Yes	Yes	Yes
Time Fixed Effects	Yes	Yes	Yes	Yes
*N*	200	195	191	200
Adjusted *R*^2^	0.785	0.667	0.560	0.809

Standard errors in parentheses, *p < 0.1, **p < 0.05, ***p < 0.01; The urban-rural disposable income gap is measured by the ratio of per capita disposable income of urban residents to that of rural residents; The urbanization level is measured by the ratio of urban population to total population; High and low groups are divided based on the median value. In economics literature, this is a normal practice. *, **, and *** represent significance levels.

## 6 Conclusions and implications

In this study, we investigate the influence of financial technology (FinTech) on regional environmental, social, and governance (ESG) performance, using China’s provincial panel data from 2011 to 2023. We reveal that FinTech significantly boosts regional ESG outcomes and fosters regional innovation. We explain theoretically on how FinTech enhances regional innovation efficiency and reduces innovation costs, thereby fostering the accumulation of regional ESG innovation capacity and ultimately improving overall ESG performance. We also find that the positive effects of FinTech are particularly amplified in regions characterized by lower income disparity and higher levels of urbanization. These findings suggest that implementing FinTech policies customized to local economic and social contexts can effectively advance ESG objectives and support sustainable regional development.

We provide implications for policymakers and firms. First, policymakers should adopt differentiated FinTech strategies that account for regional variations in income levels and urbanization to maximize ESG benefits. In order to achieve this goal, policymakers need to take a synergistic relationship between FinTech, innovation, and ESG through targeted incentives and supportive regulatory frameworks. This motivation can encourage the development of high-quality ESG data ecosystems facilitated by FinTech. Such policies might include rewarding green innovation efforts through subsidies or tax breaks, particularly for initiatives integrating digital solutions. Meanwhile, it can create a competitive market that recognizes and rewards green performance. For instance, a ‘green performance-policy dividend’ linkage mechanism could be explored to achieve this. Second, for firms, we advocate for leveraging FinTech for ESG advancement. Firms are recommended to strategically adopt FinTech solutions to enhance ESG-related innovation and improving operational efficiency in resource management, as well as to ensure transparent ESG reporting and increase access to green finance.

## Data Availability

The data analyzed in this study is subject to the following licenses/restrictions: Data will be made available on request. Requests to access these datasets should be directed to Guo Wu, guo.wu@sandau.edu.cn.

## Footnotes

^1^ https://www.chindices.com/esg-ratings.html
